# Synchronous Design of Membrane Material and Process for Pre-Combustion CO_2_ Capture: A Superstructure Method Integrating Membrane Type Selection

**DOI:** 10.3390/membranes13030318

**Published:** 2023-03-09

**Authors:** Zhiqiang Ni, Yue Cao, Xiaopeng Zhang, Ning Zhang, Wu Xiao, Junjiang Bao, Gaohong He

**Affiliations:** 1 State Key Laboratory of Fine Chemicals, Dalian University of Technology, Dalian 116023, China; 2 School of Chemical Engineering, Dalian University of Technology, Panjin 124221, China

**Keywords:** membrane separation, superstructure, pre-combustion CO_2_ capture, IGCC power plant

## Abstract

Membrane separation technology for CO_2_ capture in pre-combustion has the advantages of easy operation, minimal land use and no pollution and is considered a reliable alternative to traditional technology. However, previous studies only focused on the H_2_-selective membrane (HM) or CO_2_-selective membrane (CM), paying little attention to the combination of different membranes. Therefore, it is hopeful to find the optimal process by considering the potential combination of H_2_-selective and CO_2_-selective membranes. For the CO_2_ capture process in pre-combustion, this paper presents an optimization model based on the superstructure method to determine the best membrane process. In the superstructure model, both CO_2_-selective and H_2_-selective commercial membranes are considered. In addition, the changes in optimal membrane performance and capture cost are studied when the selectivity and permeability of membrane change synchronously based on the Robeson upper bound. The results show that when the CO_2_ purity is 96% and the CO_2_ recovery rate is 90%, the combination of different membrane types achieves better results. The optimal process is the two-stage membrane process with recycling, using the combination of CM and HM in all situations, which has obvious economic advantages compared with the Selexol process. Under the condition of 96% CO_2_ purity and 90% CO_2_ recovery, the CO_2_ capture cost can be reduced to 11.75$/t CO_2_ by optimizing the process structure, operating parameters, and performance of membranes.

## 1. Introduction

### 1.1. Background

With the rapid growth of the population and the consumption of fossil energy, the content of CO_2_ in the atmosphere is increasing year by year, which leads to the greenhouse effect and the exacerbation of global warming. The CO_2_ from flue gas and automobile exhaust is the main reason for the increase of CO_2_ concentration in the atmosphere [1]. The annual emissions from coal-fired power generation account for about 45% of the total CO_2_ emissions [2].

Pre-combustion CO_2_ capture is considered as a promising solution. Pre-combustion CO_2_ capture is usually used in integrated gasification combined cycle (IGCC) systems, having the advantages of high energy production efficiency, low pollutant emission and high fuel flexibility [3]. Because the syngas pressure is high and the CO_2_ concentration is about 40%, the higher driving force can improve the economic benefits; thus, pre-combustion CO_2_ capture is considered as the most potential CO_2_ capture method in power plants [4,5].

Generally, the absorption method (physical or chemical absorption), cryogenic separation method and membrane separation technology can be selected for pre-combustion CO_2_ capture [3]. The absorption method has the disadvantages of complicated process steps, high investment cost and high energy consumption in the regeneration process, which seriously restrict the development of this method. Membrane separation technology has many inherent advantages for CO_2_ capture, such as little impact on the environment, simple operation, and ease of scale-up and integration with other processes [6]; it is one of the most promising methods to replace absorption technology [7,8].

### 1.2. Research Progress

For the separation process of H_2_ and CO_2_ from pre-combustion CO_2_ capture, membrane types can be divided into CO_2_-selective membranes, which preferentially permeate CO_2_ (CM), and H_2_-selective membranes, which preferentially permeate H_2_ (HM). Different researchers have extensively studied CO_2_-selective membranes and H_2_-selective membranes. For CO_2_-selective membranes, the research of Franz et al. [9] showed that for pre-combustion CO_2_ capture, when the CO_2_-selective membrane with CO_2_/H_2_ selectivity of 60 was used, the CO_2_ emission of the process with a two-stage membrane cascade was greatly reduced (from 0.713kg/kWh to 0.14kg/kWh) compared with the physical absorption method. Vakharia et al. [10] analyzed the cost of CO_2_ removal from syngas by using CO_2_-selective membranes. Under the condition of 90% CO_2_ removal rate and >99% hydrogen recovery, the cost of electricity (COE) increased by 14–18% when using a two-stage membrane process, which was far lower than that of the Selexol process, at 39%. Han et al. [11] studied the process of applying CO_2_-selective membranes with different facilitating transport characteristics to a two-stage membrane system. When the recovery of H_2_ was 99%, the power cost of the membrane process was 12.5% lower than that of the Selexol process. Lin et al. [12] studied the capture process of membranes combined with cryogenic processes. The results showed that the energy consumption of CO_2_ capture in a CO_2_-selective membrane with a CO_2_/H_2_ selectivity of 10 was almost the same of that in the Selexol process; when CO_2_/H_2_ selectivity increased to 20, it could save 20% energy compared with Selexol process.

In addition to the CO_2_-selective membrane, some scholars have devoted attention to the H_2_-selective membrane. Giordano et al. [13] proposed a three-stage membrane system to separate H_2_ and CO_2_ from syngas based on an H_2_-selective membrane with a H_2_/CO_2_ selectivity of 15 and H_2_ permeance of 300 GPU. When the operating pressure was 7000 kPa, the cost of CO_2_ capture was 16.6$/tCO_2_, which was lower than that of the Selexol process and post-combustion CO_2_ capture. Krishnan et al. [14] studied the high-temperature separation system using a H_2_-selective membrane of PBI material. Compared with the separation system based on the Selexol process, the separation system based on membrane had lower energy consumption and higher net output power, because CO_2_ is obtained on the high-pressure side. However, this study also pointed out that the operating parameters of the membrane separation system were not optimized, so the advantages of the membrane system will be more obvious when optimizing parameters such as process configuration, pressure and so on.

The above-mentioned separation processes of H_2_ and CO_2_ in pre-combustion are not uniform. Membrane materials can only be given full play in proper processes. One of the most effective methods of process structure design is the superstructure method. At present, the superstructure research of pre-combustion CO_2_ capture is limited, and most of the research focuses on post-combustion CO_2_ capture.

Arias et al. [15] used mathematical programming and the superstructure-based optimization method for the first time to design the post-combustion CO_2_ capture process. In the optimization, both the optimal process and the optimal number of membrane stages were taken into account as optimization variables. Then, based on this research, Mores et al. [16] improved the model, further considering the different pressures between membrane stages and the installation of the vacuum pump on the permeate side of every membrane stage. Chiwaye et al. [17] put forward a mathematical model based on superstructure to design the process of CO_2_ capture from flue gas, allowing independent change of membrane inlet pressure at all stages, a vacuum pump and self-recycle streams. The results showed that different operating pressures at different membrane stages can effectively save the power consumption of the compressor, thus reducing the operating cost.

The above research on post-combustion CO_2_ capture was based on CO_2_-selective membranes with fixed performance. For the multi-stage membrane processes, where the membrane performance of each stage is independent and changeable, the research of Roussanaly et al. [18] proved that it is worthwhile to consider the change of membrane performance. Using membranes with different performance at different membrane stages led to a cost reduction of 7%. The research results of Gabrielli et al. [19] showed that the performance optimization of membrane materials based on each membrane stage can improve the flexibility of the system and reduce the specific membrane area and energy consumption. In Lee et al. [20], the research results of flue gas CO_2_ capture based on the superstructure method showed that when the first stage uses a membrane with high CO_2_ permeance, and the subsequent stage uses a membrane with high CO_2_ selectivity, the energy consumption and CO_2_ capture cost are reduced. Therefore, even if the same membrane type is used in different stages, the performance of the membrane needs to be changed to improve the system [21].

When considering the membrane types that preferentially permeate different gases, there is room for further reduction of CO_2_ capture cost. In response to the fact that previous research on post-combustion CO_2_ capture focused on CO_2_-selective membranes, Ren et al. [2] considered both CO_2_- and N_2_-selective membranes at the same time and compared various processes of single membranes and hybrid membranes. The results showed that, compared with single-selective membrane system, the design of a hybrid membrane can effectively reduce energy consumption and membrane area, but the permeate side of the first stage into the second stage was not considered in the two-stage process of the hybrid membrane in this study. Hussain et al. [22] designed a two-stage membrane process using N_2_- and CO_2_-selective membranes, which achieved low energy consumption (1MJ/t CO_2_) and low cost (20.5$/t CO_2_), reaching a level competitive with traditional absorption processes.

On the basis of considering the combination of membrane types, it is expected that costs will be further reduced if the membrane process design is also considered. Ohs et al. [23] optimized the superstructure model by mixed integer nonlinear programming and designed a membrane process for removing nitrogen from natural gas. The superstructure model was limited to two stages, and the effects of a nitrogen-selective membrane and methane-selective membrane on the optimal process configuration were evaluated. The results showed that compared with the cost obtained by using only one of the two types of membranes, using two types of membranes in the same two-stage process saved 40% of the process cost. In the superstructure model of flue gas CO_2_ capture proposed by Chiwaye et al. [24], both the CO_2_-selective membrane and N_2_-selective membrane were considered; at each membrane stage, there was an option of whether to preferentially permeate CO_2_ or N_2_. The results showed that compared with the optimized CO_2_-selective membrane process, the hybrid membrane process saved 46% of the total membrane area and 14% of the capture cost.

### 1.3. Aim and Novelty of This Work

According to the literature review, for pre-combustion CO_2_ capture, the advantage of using CO_2_-selective membranes is that a high-purity CO_2_ product can be obtained; however, the CO_2_ pressure is low. The advantage of using a H_2_-selective membrane is that CO_2_ is obtained from the residual side of high pressure; however, compared with using a CO_2_-selective membrane, the purity of the CO_2_ product is lower. If a CO_2_-selective membrane and H_2_-selective membrane are used at the same time, it is expected that high-purity and high-pressure CO_2_ products can be obtained at the same time, and the energy consumption and membrane area of the H_2_/CO_2_ separation system can be reduced synergistically. Therefore, as the key factors of membrane process design, the combination of various membrane types and the optimization of membrane performance are both necessary as optimization variables. At the same time, the simultaneous optimization of membrane types and membrane processes is expected to further reduce energy consumption and cost. Therefore, in order to comprehensively evaluate the potential of pre-combustion membrane separation technology, this paper puts forward the superstructure method, integrating membrane type selection, to determine the optimal membrane technology and capture cost of the pre-combustion CO_2_ capture membrane separation system. In the optimization process, both H_2_- and CO_2_-selective membranes are considered. In addition, the change of membrane permeance and selectivity according to the Robeson upper bound is also taken as the optimization variable. Aspen Hysys software is used to establish the superstructure, and the artificial ant colony algorithm is used to optimize the parameter variables at the same time to obtain the best system process.

The structure of this paper is as follows: Firstly, in the second section, the feed gas and separation requirements of pre-combustion CO_2_ capture are illustrated, and then the superstructure model, membrane performance and economic model used are described. In the third section, the feasibility analysis of the single-stage membrane is carried out, and the optimization of the following different situations is considered and compared:

(1) Optimization of two-stage membrane process based on the commercial membrane, aiming at exploring the optimal process, capture cost and operation parameters based on existing commercial membranes through parameter optimization.
(2) Using the H_2_-selective membrane changes based on the Robeson upper bound and the commercial CO_2_ membrane, the process optimization of a two-stage membrane aims to explore the potential for the reduction of the capture cost and the change of the best H_2_-selective membrane performance when the performance of H_2_-selective membrane changes.
(3) Using the H_2_-selective membrane and CO_2_-selective membrane change based on the Robeson upper bound, the optimization of a two-stage membrane process and the comparison of the optimal process of membrane type combination aim at determining the influence of two membrane types on the optimal process and its capture cost; the necessity of using a hybrid membrane is clarified by analyzing the gap between different membrane type combinations.

## 2. Model and Optimization Method

### 2.1. Problem Formulation

In the IGCC system, coal is used as the raw material; after pressurized gasification, it becomes syngas rich in H_2_ and CO. The syngas firstly removes the particles, and then CO and H_2_O react to generate CO_2_ and H_2_ through water–gas shift (WGS). The equation is shown in formula (1). For the CO_2_ capture unit of IGCC system, CO_2_ is obtained before combustion. H_2_S and H_2_O are removed from the syngas, then CO_2_ is removed in the membrane separation unit, and the remaining H_2_ enters the gas turbine to generate electricity.

(1) $$\mathrm{CO}+H_{2}O\to {\mathrm{CO}}_{2}+H_{2}\times \Delta h=-40.6\;\mathrm{kJ}/\mathrm{mol}$$

The scale of the IGCC power plant referred in this paper is 556 MW. The syngas flow rate is 28,390 kmol/h, while the pressure and temperature are 3000 kPa and 40 ℃, respectively. The composition of syngas is 60% H_2_ and 40% CO_2_ [25,26].

The CO_2_ capture unit needs to reduce the loss of H_2_ as much as possible while achieving the goal of CO_2_ separation. Most studies take 90% CO_2_ recovery as the basic goal. In order to ensure power generation, H_2_ products should be controlled at 3000 kPa. In order to facilitate the storage and transportation of CO_2_ products, the CO_2_ purity should be above 96%. CO_2_ needs to be compressed to a low-temperature liquid state. At the same time, in order to reduce the loss of power generation, the purity of H_2_ in CO_2_ should not exceed 4% [13].

### 2.2. Mathematical Modeling

The polymer membrane is the most industrialized membrane type at present because of its moderate price and easy large-scale manufacture. The solution-diffusion mechanism is considered to better describe the process of the permeation of multi-component gas mixtures through polymer membrane. Various mathematical models of gas separation membranes based on the solution-diffusion mechanism have been widely studied.

**Figure 1:**
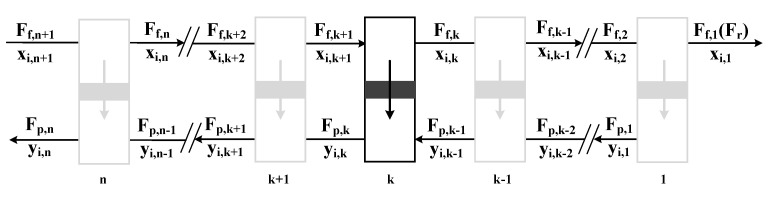
Schematic diagram of membrane unit model (counter-current).

Weller and Steiner [27] first put forward a mathematical model for membrane separation of binary gas mixtures, and Pan et al. [28,29] proposed and optimized a model suitable for separating multiple components and put forward a “shooting method” to solve it. However, it is difficult to obtain an accurate value in the case of high stage cut or low raw material flow rate/membrane area ratio. Based on previous research, Coker et al. [30] discretized the whole membrane and decomposed it into multiple infinitesimals to optimize the solution method, which can be simulated at a high stage cut. In this paper, according to the mathematical method proposed by Coker et al. [30], the membrane unit model is established, and it is assumed that the membrane unit operates based on a counter-current flow pattern in the form of hollow fibers [31], as shown in Figure 1. The membrane module is divided into 100 equal area increments, and the permeability of component *i* in a stage is calculated by the following formula:

(2) $$m_{i,k}=Q_{i}\Delta A_{k}\left(P_{F_{p,k}}x_{i,k}-P_{F_{p,k}}y_{i,k}\right)$$

where *m_i,k_* is the molar flow rate (kmol/s) of component j through the kth stage, *Q_i_* is the permeance of the membrane to gas component *i* (kmol/(m^2^s·Pa)), *F_f,k_* and *F_p,k_* represent the flow rates of the feed side and permeate side of the membrane at the kth stage, *x* and *y* are the compositions of component *i* in the feed side and permeate side, respectively, and *P_Ff,k_* and *P_Fp,k_* are the pressure (Pa) on the feed side and the permeate side, respectively.

The established model includes the following assumptions:

(1) There is no pressure loss between feed and residual sides [17,32,33].
(2) The membrane is operated at an isothermal temperature [33].
(3) The phenomenon of concentration polarization is neglected [17,32].
(4) The permeance and selectivity are constant [32,33].
(5) The solution-diffusion model is used to describe the membrane transport intrinsic properties [20,33].

The construction of the membrane unit model first needs to compile the mathematical model of the membrane operation unit, compile the model into DLL (dynamic link library) file, and then register it in Aspen Hysys software before it can be used.

### 2.3. Superstructure Model and Optimization Method

At present, the research on multi-stage membrane focuses on two to four stages. The research of Hao et al. [34] showed that the process of a two-stage membrane is more economical than that of a three-stage membrane. Ramírez et al. [33] developed a nonlinear programming model based on a four-stage membrane superstructure. For high recovery and separation targets, the CO_2_ capture cost of the three-stage membrane separation process is only slightly lower than that of the two-stage configuration, but the complexity of the three-stage membrane separation process is greatly increased. The difference between the four-stage and the three-stage configuration is negligible. The research results of Datta et al. [35] showed that there is no obvious difference between the optimal process of the two-stage membrane and three-stage membrane in most cases. Therefore, the two-stage membrane can take into account the complexity of process and separation performance. This paper considers the two-stage membrane separation process.

**Figure 2:**
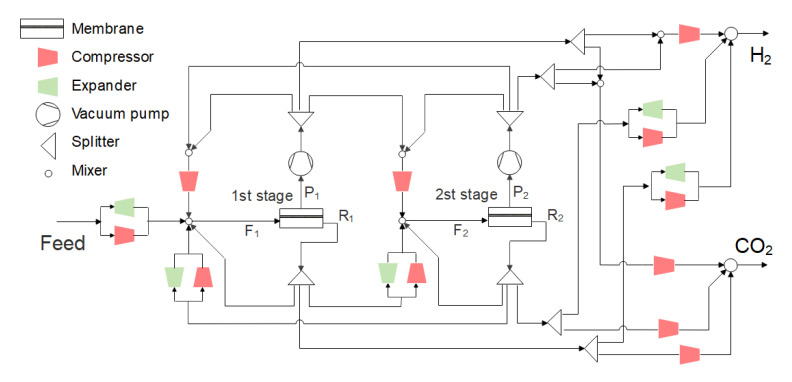
Superstructure model of two-stage membrane process.

The superstructure of the two-stage membrane separation process proposed in this paper is shown in Figure 2. The feed gas F_1_ first enters the first stage. Driven by the pressure difference between the feed side and the permeate side, the gas passing through the membrane is called permeate side stream P_1_, and the gas not passing through the membrane is called the residue side stream R_1_. P_1_ and R_1_ streams can be recycled to the inlet of the first stage or used as the feed side F_2_ of the second stage, or as a H_2_ or CO_2_ product. Similarly, after the feed side F_2_ enters the second stage membrane unit, the permeate side stream P_2_ and the residue side stream R_2_ of the second stage membrane unit can be recycled to the inlet of the second stage membrane unit as the feed side F_2_ or returned to the first stage membrane unit as the feed side F_1_, and can also be used as a H_2_ or CO_2_ product. The process structure is controlled by changing the split ratio of the splitter. It is the pressure difference that decides whether the compressor or expander is selected (the compressor is selected when the pressure difference is greater than 0, and the expander is selected when the pressure difference is less than or equal to 0).

In order to ensure that the hydrogen entering the gas turbine reaches the specified pressure, multi-stage compression of H_2_ and CO_2_ products and the cooler after the compression are installed in the superstructure (the multi-stage compression in the superstructure is represented by a single compressor in Figure 2). The H_2_ product is compressed to 3000 kPa after mixing, and the CO_2_ product is cooled to 10 ℃ after multi-stage compression to 15,000 kPa. The isentropic efficiency of compressor, vacuum pump and expander is specified as 75% [36].

When considering the change of membrane materials, the typical Mixed-Integer Nonlinear Programming (MINLP) problem is addressed to complete the membrane process design by establishing a superstructure model and optimizing its calculation, which can be expressed by the following formula:

(3) $$minF\left(x\right)$$

(4) $$\mathrm{st}.\left\{\begin{array}{c} h_{i}\left(x\right)=0,\forall i\\ purity=0.96;\;0.97;\;0.98;\;0.99\\ recovery=0.90;0.92;0.94;0.96;0.98 \end{array}\right.$$

where *f*(*x*) is the objective function in the above formula. In this paper, *f*(*x*) is the CO_2_ capture cost. The calculation method is explained in detail in the Section 2.5. *h*(*x*) is the equilibrium equality constraint of mass and energy in the model, and *x* is the vector of model variables. The setting of the optimization parameters is key to realizing efficient operation. The optimization parameters are divided into three parts: the selection of membrane types and their performance, the process structure and the system operation parameters, as shown in Table 1.

Optimization variables include binary discrete variables and continuous variables. Binary variables mainly determine the selection of membrane material type or process structure. In this paper, we define them as membrane type selection coefficients (x_1_ and x_2_), recycle stream selection coefficients (x_7_ and x_13_) and selection coefficients of the stream going to another membrane stage (x_9_ and x_11_), respectively. These coefficients are all integer variables (0 or 1 in this paper), but different coefficients have different meanings. Membrane type selection coefficient x_1_ and x_2_ determine the membrane type of each stage, with the value of 1 representing a H_2_-selective membrane and 0 representing a CO_2_-selective membrane. Recycle stream selection coefficient x_7_ and x_13_ determine the recycle stream of each stage, with the value of 1 representing the permeate side and 0 representing the residual side. Selection coefficients of the stream going to another membrane stage x_9_ and x_11_ determine the stream to the another stage, with a value of 1 representing the permeate side and 0 representing the residue side. Continuous variables include selectivity of each stage (x_3_ and x_4_), membrane area (x_5_ and x_6_), recycle percentage (x_8_ and x_14_), flow percentage of the stream going to another stage (x_10_ and x_12_) and inlet/outlet pressure of membrane (x_15_~x_18_). The range of membrane inlet pressure is set to be 105–5000 kPa, and the range of the membrane permeate side pressure is set to be 20–105 kPa [37]. The membrane area of each stage ranges from 500 m^2^ to 200,000 m^2^. When the membrane type changes, the model automatically sets the temperature of the membrane inlet stream to the operating temperature required for membrane.

In order to improve the solution efficiency, some infeasible situations are excluded in the process parameter setting, including:

(1) Avoid the permeate/residue side stream of each stage membrane as the feed gas of another stage membrane at the same time, because this is actually the reverse process of separation [20].
(2) Try to avoid concentration mixing between membrane stages. If both the first-stage and the second-stage membranes are H_2_-selective or CO_2_-selective membranes, the stream of the second-stage membrane returning to the first-stage membrane will be opposite to the stream of the first-stage membrane going to the second-stage membrane. If the first-stage and second-stage membranes are different, the stream from the second-stage membrane back to the first-stage membrane must be the same as that from the first-stage membrane to the second-stage membrane.

The superstructure model is built by Aspen Hysys software, and the Peng-Robinson equation is used to calculate thermodynamic properties. Use the method of an internal spreadsheet to realize the connection process with external software to process all data types at the same time. Choose Matlab tools to exchange data with Aspen Hysys by ActiveX to realize the optimization of process. The artificial ant colony algorithm is used by Matlab to optimize the superstructure model. After the optimization, the membrane types, their performance of each stage of membrane, the best membrane process and the corresponding system operation parameters can be obtained.

### 2.4. Commercial Membrane Performance and its Robeson Upper Bound

In the membrane separation process, different gas components are separated by the difference of permeability, and the multiple of fast gas relative to slow gas is expressed by selectivity *α*. First, in order to clarify the cost level that the existing commercial membrane can achieve, the commercial membrane of MTR is selected [4]; the performance of the mixed-gas membrane is shown in Table 2. The H_2_-selective membrane needs to operate at a high temperature (150 °C) for appreciable permeance and selectivity; CO_2_-selective membranes have better selectivity at a cold temperature (10 °C).

There is a trade-off between permeance and selectivity for polymer membranes in most separation systems. In order to study the best trade-off point of membrane performance, the membrane performance will be allowed to change within a certain range. This is an optional method to quantify the relationship between permeance and selectivity with the Robeson upper bound.

In 2008, Robeson et al. [38] reviewed the relationship of the permeability and selectivity values of polymer membrane materials in order to establish a trade-off and proposed that the relationship can be calculated according to the formula $P_{i}=k\times \alpha_{ij}^{n}$. For an H_2_-selective membrane, *k* = 4515 and *n* = 2.302. For a CO_2_-selective membrane, the relationship between the permeability and selectivity is studied with reference to the research of Han et al. [39], where *k* = 0.0194 and *n* = −4.0178.

It is worth noting that additional types of membrane materials with excellent performance have been developed [40], such as metal membranes [41], ceramic membranes [42], nano-porous monolayer graphene membranes [43], and facilitated transport membranes [39], which are far superior to those membranes with the Robeson upper bound. It is expected that the membrane process will be competitive by further improving the membrane performance; however, this paper does not discuss this, instead devoting attention to the influence of the performance change of commercial polymer membrane on the process cost.

### 2.5. Economic Model and Performance Parameters

The specific calculation of the economic model is shown in Table 3. The investment considers membrane, compressor, vacuum pump, expander and heat exchanger equipment, in which it is assumed that the life of the membrane is 5 years and that of other equipment is 25 years. The price of membranes used in this paper is 240$/m^2^ [44]. Operation and maintenance costs include membrane and equipment replacement and maintenance costs, in which the membrane module maintenance cost is 1% of its investment cost, the other equipment cost is 3.6% of the purchase cost, and the electricity cost is calculated by compressor and vacuum pump power consumption minus expander power consumption multiplied by annual running time (8000 h) and electricity cost (0.05$/kWh). Compared with the cost of the membrane, compressor and electricity, the cost of the heating steam and cooling water is negligible, so it does not appear in the cost calculation formula.

The objective of the paper is to accomplish automated design by a superstructure simulation integrating membrane type selection. Taking the CO_2_ capture cost as the objective function, the specified purity and recovery are calculated by the following formula:

(5) $$Purity=\frac{V_{production,CO_{2}}\times X_{production,CO_{2}}}{V_{production,CO_{2}}}$$

(6) $$Recovery=\frac{V_{production,CO_{2}}\times X_{production,CO_{2}}}{V_{feed}\times X_{feed,CO_{2}}}$$

where *V* and *X* are molar flow rate and molar composition. Subscript feed is the feed of the separation system.

The membrane area and power consumption per ton CO_2_ product are expressed by specific membrane area and specific power consumption:

(7) $$A_{\mathrm{spe}}=\frac{\mathrm{Total}\;\mathrm{membrane}\;\mathrm{area}}{V_{\mathrm{production},CO_{2}}\times X_{\mathrm{production},CO_{2}}}$$

(8) $$W_{\mathrm{spe}}=\frac{W_{\mathrm{net}}}{V_{\mathrm{production},CO_{2}}\times X_{\mathrm{production},CO_{2}}}$$

The energy consumption of the system is calculated by Formula 9:

(9) $$W_{\mathrm{net}}=W_{\mathrm{com}}+W_{\mathrm{vp}}-W_{\mathrm{ex}}$$

Taking the average capture cost as the objective function, the purity and recovery are limited by the penalty function, and the formula is as follows:

(10) $$F\left(x\right)=Cost+r\times ({\left(Pu{\mathrm{rity}}_{calculate}-Pu{\mathrm{rity}}_{t\arg et}\right)}^{2}+{\left(Rec{\mathrm{overy}}_{calculate}-Rec{\mathrm{overy}}_{t\arg et}\right)}^{2}).$$

where *F*(*x*) is the objective function, Cost is the capture cost ($/t CO_2_), *r* is the penalty factor, and the value is 1 × 10^8^. The lower corner “*target*” is the specified purity and recovery.

## 3. Results and Discussion

### 3.1. Feasibility Analysis of Single-Stage Membrane System

**Figure 3:**
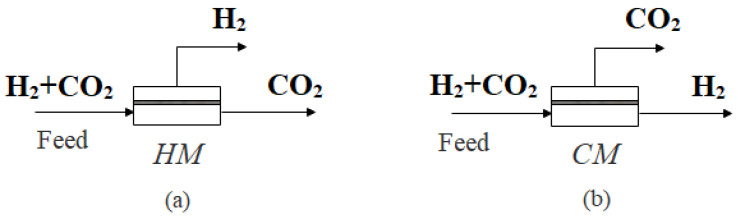
Schematic diagram of single-stage membrane separation. (**a**) H_2_-selective membrane; (**b**) CO_2_-selective membrane.

In order to clarify the separation effect of the single-stage membrane process, this section simulates and analyzes the separation of H_2_/CO_2_ in the single-stage membrane process. For H_2_/CO_2_ separation, either H_2_- or CO_2_-selective membranes can be selected, as shown in Figure 3. The feed gas is composed of 60% (mole fraction) H_2_ and 40% (mole fraction) CO_2_; the CO_2_ product is required to reach 96% purity and 90% recovery.

**Figure 4:**
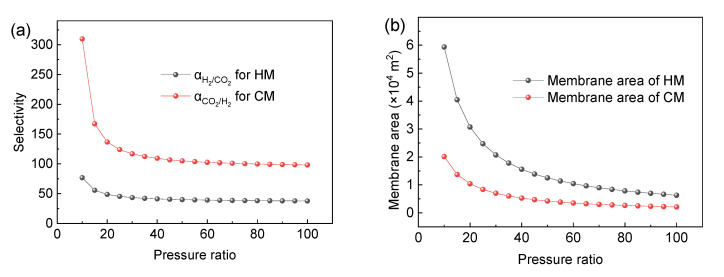
The selectivity (**a**) and membrane area (**b**) required of HM and CM under different compression ratios with CO_2_ purity and recovery set to 96% and 90% (assuming H_2_ permeance of CO_2_-selective membrane and CO_2_ permeance of H_2_-selective membrane are 20 GPU).

When the CO_2_ product is required to reach 96% purity and 90% recovery, the required selectivity *α* of H_2_ and the CO_2_-selective membrane under different pressure ratios is determined, as shown in Figure 4a. As can be seen from Figure 4a, when the pressure ratio increases, the required selectivity of membrane decreases and tends to be stable. As the pressure ratio increases, the driving force of the membrane separation process increases, so the required selectivity decreases under the same separation requirement.

For the H_2_-selective membrane, no matter how large the pressure ratio is, the selectivity needs to be close to 50 to achieve 96% CO_2_ purity and 90% CO_2_ recovery. For the CO_2_-selective membrane, the selectivity should be close to 100 to achieve 96% CO_2_ purity and 90% CO_2_ recovery. However, it is difficult for commercial polymer membranes to achieve such high selectivity at present.

**Figure 5:**
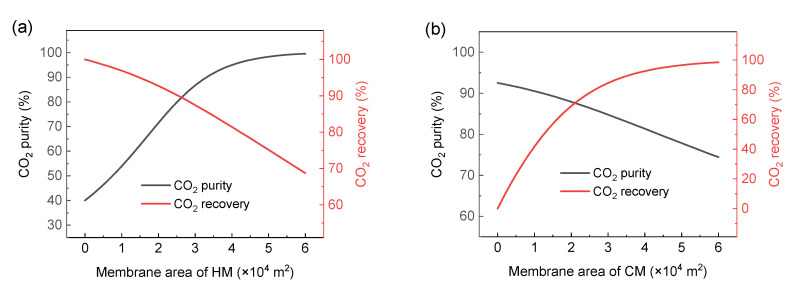
The relationship between the purity and recovery of CO_2_ under the condition of pressure ratio of 30 and selectivity of 20 when membrane area changes (**a**) the change of the membrane area of HM (**b**) the change of the membrane area of CM.

At the same time, it can also be seen from Figure 4 that the selectivity required by the CO_2_-selective membrane is obviously larger than that of the H_2_-selective membrane under the same pressure ratio and separation requirement. The reason is that when the CO_2_ purity is required to be 96%, in order to ensure that CO_2_ reaches such high purity in the CO_2_-selective membrane, it is necessary to maintain a small membrane area, as shown in Figure 4b. At the same time, in order to ensure 90% CO_2_ recovery, the system can only meet the requirements of purity and recovery by greatly increasing the selectivity. Therefore, for the separation requirement of high CO_2_ concentration, the selectivity of the CO_2_-selective membrane is higher than that of the H_2_-selective membrane. It can be seen from the Robeson upper bound that it is difficult for the selectivity of the H_2_-selective membrane or the CO_2_-selective membrane to reach more than 20, while it can be seen from Figure 5 that the pressure ratio above 30 has little effect on the system performance.

Therefore, the variation of CO_2_ purity and CO_2_ recovery with the membrane area is studied under the condition of a pressure ratio of 30 and selectivity of 20, as shown in Figure 5. It can be seen that there is an obvious trade-off relationship between the purity and the recovery when the membrane area changes. When the CO_2_ purity is above 96%, the recovery above 90% cannot be achieved. When the recovery is high, it is difficult for the purity to reach above 96%.

For example, for the H_2_-selective membrane, the purity of the CO_2_ product can reach more than 96%, but the highest CO_2_ recovery can reach about 80% at this time. When it comes to achieving 90% CO_2_ recovery, the CO_2_ purity can only reach 80%.

For the CO_2_-selective membrane, the highest CO_2_ purity can only reach 92.7%, and the CO_2_ recovery is close to 0 at this time. When it comes to achieving a CO_2_ recovery of more than 90%, the purity of CO_2_ can only reach about 80%. Obviously, it is difficult to achieve the ideal separation requirements using a single-stage membrane process for the current industrialized membrane materials. This is consistent with some previous research results. Havas et al. [47] put forward that because of the trade-off between selectivity and permeability, the single-stage membrane design is simple in process, but the lack of a recycle stream leads to a huge loss of product, and the high recovery cannot be guaranteed on the premise of ensuring purity. Han et al. [11] also pointed out that a non-reactive polymer membrane is not suitable for the single-stage membrane process. The reason is that the present polymer membrane has difficulty achieving such high selectivity; even if it can achieve high selectivity, it must sacrifice the permeability, thus limiting the simultaneous realization of purity and recovery. Therefore, in order to achieve the required separation requirements, at least a two-stage membrane separation process must be adopted.

### 3.2. Optimization of Membrane Process Based on Commercial Membrane

According to the literature review, most previous scholars’ research focused on a single membrane type. For a H_2_/CO_2_ separation system, when an H_2_-selective membrane is used, high-concentration H_2_ is obtained from the low-pressure permeate side of membrane, and the CO_2_ product is obtained from the high-pressure residue side. On the contrary, when a CO_2_-selective membrane is used, the CO_2_ product is obtained at atmospheric pressure, and the H_2_ product is obtained at high pressure. When the CO_2_/H_2_ product has a high-pressure requirement, the product can be obtained from the residual side by reasonably designing the combination of different membrane types, thus saving the compression energy consumption.

#### 3.2.1. Optimization of Two-Stage Membrane Process Considering Membrane Type Combination When the Operating Pressure of Each Stage Is Fixed

**Figure 6:**
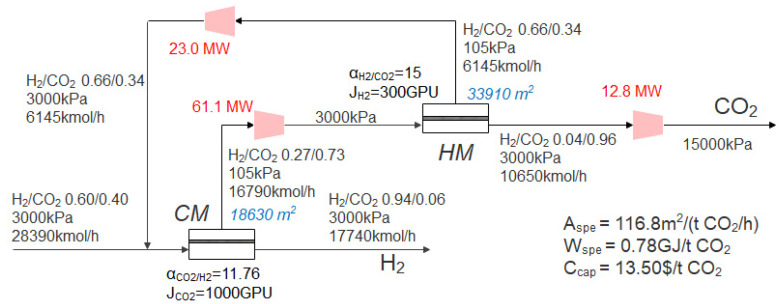
The optimal process when using the commercial membrane in Table 2, with feed pressure fixed at 3000 kPa.

Because the pressure of the syngas is relatively high, the syngas can directly enter the membrane by its own pressure. Firstly, the optimal process is studied when the CO_2_ product requires 96% purity and 90% recovery under the condition of a fixed operating pressure of 3000 kPa. The superstructure model shown in Figure 2 is adopted, and the CO_2_ capture cost is taken as the objective function for optimization. The optimization results show that the optimal process is a CM+HM two-stage process with a recycle stream, as shown in Figure 6. Firstly, the feed gas enters the CO_2_-selective membrane, and the H_2_ concentration in the feed gas is high (60%), so the H_2_-rich stream on the residue side of the first stage CO_2_-selective membrane contains 94% of H_2_ as the H_2_ product. The permeated CO_2_-rich gas is pressurized to 3000 kPa and then enters the H_2_-selective membrane. Similar to the first-stage CO_2_-selective membrane, the gas containing 96% CO_2_ on the residue side of the H_2_-selective membrane is cooled to 10 ℃ after the three-stage compression and sent out as a CO_2_ product, while the gas on the permeate side of the H_2_-selective membrane contains 66% H_2_, which is pressurized and returned to the inlet of the first-stage membrane to be mixed with feed gas. The CO_2_ capture cost of this process is 13.50 $/t CO_2_. This is basically consistent with the conclusion of Lin et al. [12] that the cost of producing liquid CO_2_ by membrane separation technology is in the range of 15–20 $/t CO_2_ in pre-combustion CO_2_ capture.

Although the self-recycle process of each stage in the superstructure model is set up, there is no self-recycle in the optimal process in Figure 6. Some studies also think that the self-recycle between single membrane stages does not play a role or has limited improvement on the process, and thus is not considered [48]. On the other hand, it is necessary to return a part of the permeate side stream of the second-stage membrane to the first-stage membrane, which makes it easier to achieve the goal of purity and recovery at the same time compared with the process without recycle [19]. Considering the molar flow rate and product composition of the stream, it is not suitable to be directly used as a H_2_/CO_2_ product.

At the same time, the vacuum pump does not appear in the optimal process. The reason is that, on the one hand, the feed gas pressure is high, and no extra vacuum pump is needed to increase the driving force, so the CO_2_ product is at a high pressure, and the use of a vacuum pump will increase the compression energy consumption. On the other hand, as shown in Table 3, compared with the compressor, the cost of a vacuum pump is higher. These factors lead to the absence of a vacuum pump in the optimal process, which is also different from the existence of a vacuum pump in the optimal process of post-combustion [20,49]. Different from the pre-combustion CO_2_ capture process, the pressure of the flue gas in post-combustion CO_2_ capture is close to atmospheric pressure, so the combination of a compressor and vacuum pump can improve the driving force of the membrane separation process more efficiently. At the same time, the N_2_ stream and CO_2_ products are in a low-pressure state in the literature, and the use of a vacuum pump on the membrane permeate side can also save the investment cost of the expander caused by product depressurization.

#### 3.2.2. Optimization of Operating Parameters of the Two-Stage Membrane Process

**Figure 7:**
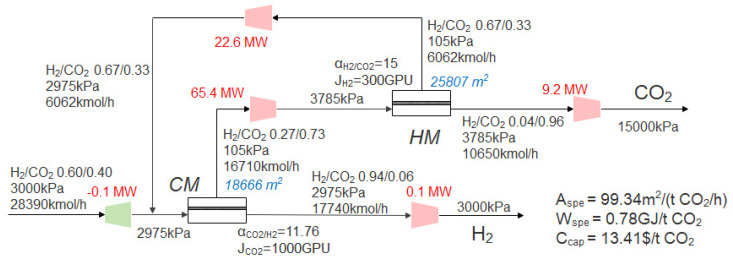
The optimal process when using the commercial membrane in Table 2 and optimizing the pressure.

For the two-stage membrane process using a commercial membrane, when the operating pressure of each stage of the membrane is optimized, it is expected to further reduce the CO_2_ capture cost by the trade-off between the power consumption and membrane area. When CO_2_ product requires 96% purity and 90% recovery, the superstructure model shown in Figure 2 is also adopted, and the CO_2_ capture cost is taken as the objective function for optimization. The optimization results show that the optimal process is still the CM+HM two-stage process, as shown in Figure 7.

Compared with the fixed operating pressure of each stage of the membrane, under the condition of 96% purity and 90% recovery of the CO_2_ product, the expander and compressor are added on the feed side and the product side respectively, and the inlet pressure of the first-stage membrane decreases slightly (from 3000 kPa to 2975 kPa). Although the power consumption of the expander and compressor is basically the same, the power consumption of the feed compressor is reduced when the second-stage membrane permeate side returns to the first-stage membrane feed side, where the feed pressure is reduced. At the same time, as the inlet pressure of first-stage membrane decreases, the membrane area increases slightly due to the reduction of driving force.

At the same time, after the pressure optimization, the inlet pressure of the second-stage membrane in the optimal process increases from 3000 kPa to 3785 kPa. The increase of driving force leads to the decrease of the second-stage membrane area, which leads to the decrease of the total membrane area from 52,540 m^2^ to 44,473 m^2^. Although the increase of the second-stage membrane inlet pressure leads to the increase of the power consumption of the second-stage membrane feed compressor (from 61.1 MW to 65.4 MW), the increase of the second-stage membrane residue side pressure also reduces the compression power consumption of the CO_2_ product on the second-stage membrane residue side, so the specific power consumption of the system is basically the same as the one with fixed membrane inlet pressure of 3000 kPa, and the system cost is reduced from 13.50 $/t CO_2_ to 13.41 $/t CO_2_.

**Figure 8:**
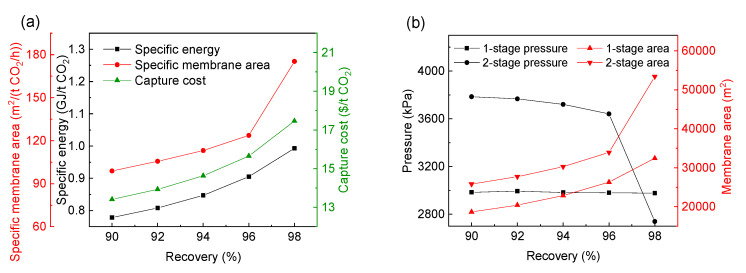
Effect of increasing CO_2_ recovery on the performance index and the best operating parameters of the optimal process using the commercial membrane in Table 2. (**a**) The CO_2_ capture cost, specific power consumption and membrane area; (**b**) optimal pressure and area of each membrane.

Figure 8 shows the changes of the performance index and the best operating parameters of the optimal process when the CO_2_ purity is 96% and the CO_2_ recovery is in the range of 90–98%. It can be seen from Figure 8a that with the increase of CO_2_ recovery, the CO_2_ capture cost, specific power consumption and membrane area gradually increase, and the CO_2_ capture cost increases from 13.41 $/t CO_2_ at 90% CO_2_ recovery to 17.46 $/t CO_2_ at 98% CO_2_ recovery. The specific membrane area increases more obviously than the specific power consumption. It can be seen from Figure 8b that with the increase of CO_2_ recovery, the inlet pressure of the first-stage membrane in the optimal process is always close to the inlet pressure of 3000 kPa, but the second-stage pressure gradually decreases from 3785 kPa to 2740 kPa, and the membrane area of each stage gradually increases. As the membrane process is mainly determined by the membrane investment cost, compression investment and power consumption cost [50], the lowest cost requires the balance between membrane operating pressure and membrane area [51]. Therefore, there must be an optimal pressure to minimize the total cost by balancing the investment cost and power consumption cost. Yang et al. [46] and Yuan et al. [52] have also drawn similar conclusions in the research of membrane separation technology in post-combustion CO_2_ capture.

**Figure 9:**
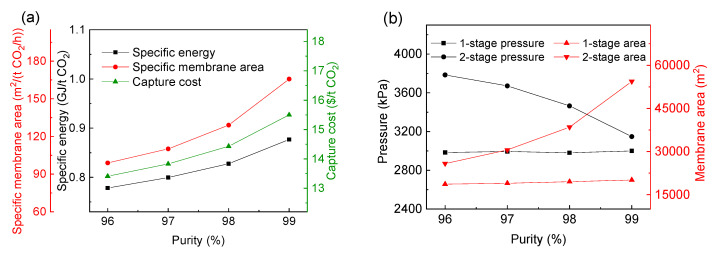
Effect of increasing CO_2_ purity on the optimal process using the commercial membrane in Table 2. (**a**) CO_2_ capture cost, specific power consumption and membrane area; (**b**) optimal inlet pressure and area of each stage membrane.

Figure 9 shows the changes of the optimal process performance index and the best operating parameters when the CO_2_ recovery is 90% and the CO_2_ purity varies from 96% to 99%. It can be seen from Figure 9a that with the increase of purity, the specific power consumption, specific membrane area and CO_2_ capture cost gradually increase. It can be seen that compared with high purity, high recovery requires a higher cost. As can be seen from Figure 8 and Figure 9, the CO_2_ capture cost of 99% CO_2_ purity is about 15.5 $/t CO_2_, while the recovery of 98% requires 17.46 $/t CO_2_.

With the increase of purity, as shown in Figure 9b, the pressure of the first-stage membrane in the optimal process is basically unchanged, but the area of the first-stage membrane increases slightly due to the increase of the recycle flow rate. While the area of the second-stage membrane gradually increases, the inlet pressure of the second-stage membrane gradually decreases. However, because the increase of the second-stage membrane area leads to the increase of the recycle flow rate (permeate side of second-stage membrane) in the system, the power consumption of the system still increases gradually.

### 3.3. Optimization of Membrane Process Based on Robeson Upper Bound

In Section 3.2, commercial membranes are used for process design, and the separation performance of membranes is fixed. According to the literature review, even for the same type of membrane, different stages of the membrane may require different separation performance. In order to explore the minimum CO_2_ capture cost of the process, this section considers variation of the permeance and selectivity based on the Robeson upper bound of different types of membranes (H_2_-selective and CO_2_-selective membranes).

It is worth pointing out that by including the correlation formula between permeance and selectivity in the Robeson upper bound in the optimization variable, the potential ideal membrane performance can be determined based on the existing commercial membrane. This may not necessarily exist in current membrane materials or may not yet have reached the commercialization level, but it can provide effective guidance for the targeted development of new membrane materials. Reasonable collocation of membrane performance, process and process variables can maximize the advantages of low energy consumption of membrane technology.

In the H_2_-selective membrane, the best performance can be determined by optimization because of the trade-off between permeability and selectivity. However, because the permeability and selectivity in the CO_2_-selective membrane increase or decrease synchronously, it is obvious that the higher the performance of the CO_2_-selective membrane material, the lower the capture cost. In order to distinguish the contribution of H_2_-selective and CO_2_-selective membranes to cost change, the following section is divided into two parts:

First, the commercial CO_2_-selective membrane with fixed performance is selected, while the performance of the H_2_-selective membrane changes based on the Robeson upper bound.

Second, both the H_2_-selective membrane and CO_2_-selective membrane change based on the Robeson upper bound.

#### 3.3.1. Optimization of H_2_-Selective Membrane Performance

**Figure 10:**
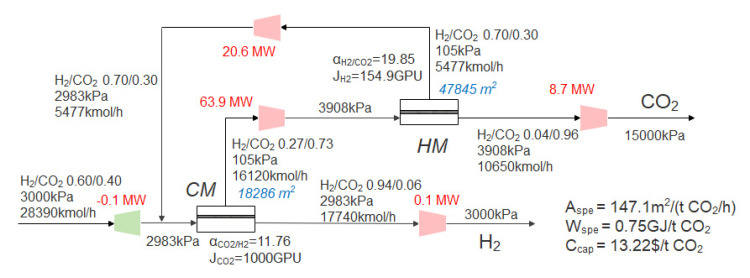
The optimal process when the performance of the H_2_-selective membrane changes based on the Robeson upper bound.

Based on the Robeson upper bound, the selectivity of HM varies from 2 to 30, and the commercial CO_2_-selective membrane in Section 3.2 is still used. When the CO_2_ purity is 96% and the recovery is 90%, optimization takes the CO_2_ capture cost as the objective function by the superstructure model shown in Figure 2. The optimal process obtained is shown in Figure 10; the CM+HM two-stage process is still the optimal process. Compared with Figure 7, the optimal H_2_-selective membrane selectivity increased from 15 to 19.85, and the permeance decreased from 300 GPU to 154.9 GPU. Compared with Figure 7, the optimal second-stage membrane pressure of the system in this section is increased from 3785 kPa to 3908 kPa, because the advantages of high selectivity need to be reflected in higher pressure ratio, while the advantages of higher permeability are more obvious at lower pressure ratio [53]. The decrease of the permeance of the H_2_-selective membrane leads to the increase of the total membrane area (44,473 m^2^ to 66,131 m^2^) in the optimal process, while the increase of the H_2_-selective membrane selectivity reduces the molar rate of recycle flow, thus reducing the total power consumption (97.2 MW to 93.2 MW). In this section, through the performance optimization of the H_2_-selective membrane, although the membrane investment cost is increased, the power consumption cost is reduced at the same time, resulting in further reduction of CO_2_ capture costs, from 13.41 $/t CO_2_ to 13.22 $/t CO_2_.

**Figure 11:**
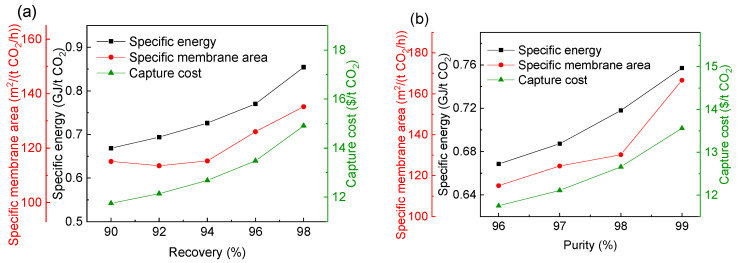
Effect of increasing CO_2_ recovery (**a**)and purity (**b**) on the CO_2_ capture cost, specific power consumption and membrane area in the optimal process.

With the increase of CO_2_ recovery and purity, the information on the optimal process, including the changes of CO_2_ capture cost, the specific power consumption and the membrane area are determined, as shown in Table 4, Table 5 and Figure 11, respectively. It can be seen that with the increase of CO_2_ recovery and purity, the CO_2_ capture cost, specific power consumption and membrane area of the optimal process all gradually increase. At the same time, it can be seen from Table 4 and Table 5 that the optimal selectivity of the H_2_-selective membrane has been kept at about 20. Therefore, the selection of the H_2_-selective membrane materials for this process should be further studied to increase selectivity.

#### 3.3.2. The Performance of H_2_-Selective Membrane and CO_2_-Selective Membrane Changing Simultaneously

In this section, the performance of the H_2_-selective membrane and CO_2_-selective membrane change at the same time. Considering the possible range of the Robeson upper bound, the selectivity range of the H_2_-selective membrane is 2–30, and that of the CO_2_-selective membrane is 5–15.

**Figure 12:**
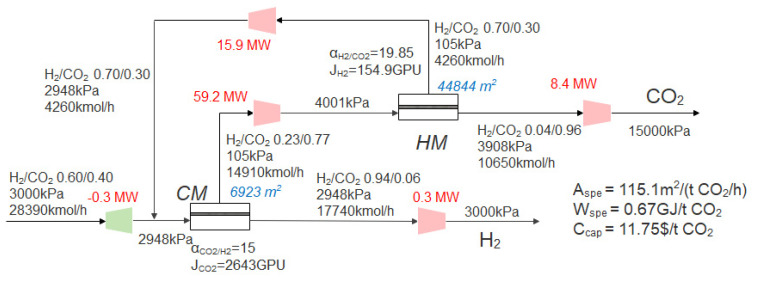
The optimal process when the performance of the H_2_-selective membrane and CO_2_-selective membrane changes based on the Robeson upper bound.

When the CO_2_ purity is 96% and the CO_2_ recovery is 90%, optimization takes the CO_2_ capture cost as the objective function by the superstructure model shown in Figure 2. The optimal process is shown in Figure 12. The positive slope of the Robeson upper bound of CO_2_ permeability and selectivity shows that high CO_2_ permeability and high CO_2_/H_2_ selectivity can be achieved at the same time, so the upper bound of the selectivity range of the CO_2_-selective membrane with both high permeance and selectivity (selectivity of 15, permeance of 2643 GPU) is the best CO_2_-selective membrane performance. For the H_2_-selective membrane, there is a trade-off relationship between permeability and selectivity. There is a compromise between high permeability and high selectivity. The optimization result shows that the optimal permeance is 140.6 GPU, and the selectivity is 20.7. Compared with the previous section (the CO_2_-selective membrane changes based on the commercial membrane, and the H_2_-selective membrane changes based on the Robeson upper bound), the total membrane area is reduced from 66,131 m^2^ to 51,767 m^2^, the total power consumption is reduced from 93.2 MW to 83.5 MW, and the CO_2_ capture cost is reduced from 13.22 $/t CO_2_ to 11.75 $/t CO_2_ due to the improvement of permeability and selectivity.

With the increase of CO_2_ recovery or purity, the change of the CO_2_ capture cost, specific power consumption and membrane area of the optimal process are determined, as shown in Table 6 and Table 7. With the increase of CO_2_ recovery or purity, the CO_2_ capture cost, specific power consumption and membrane area all increase gradually. At the same time, the best selectivity of the CO_2_-selective membrane is always 15, and the best selectivity of the H_2_-selective membrane is kept at about 20.

### 3.4. Influence of the Combination of the Membrane Type

**Figure 13:**
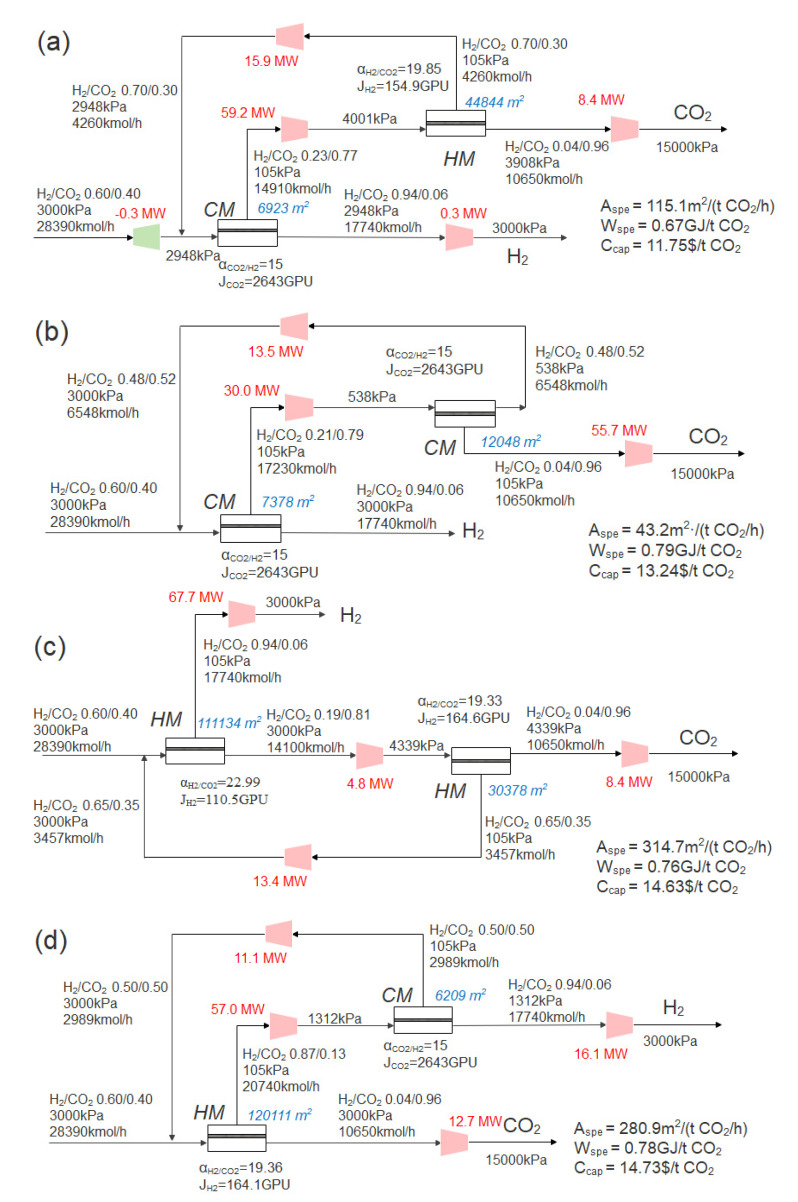
Optimal process of two-stage membrane type combination under the condition of 96% CO_2_ purity and 90% CO_2_ recovery. (**a**) CM+HM; (**b**) CM+CM; (**c**) HM+HM; (**d**) HM+CM.

**Figure 14:**
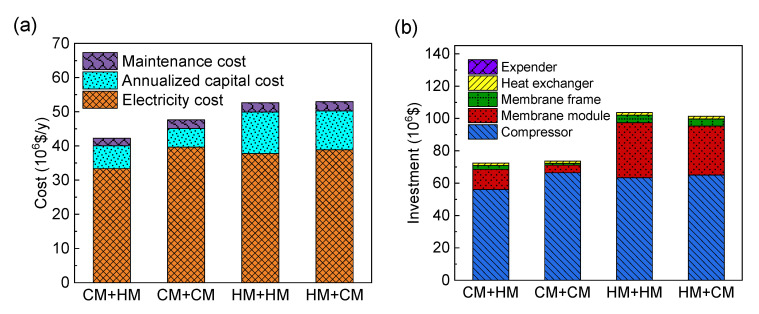
(**a**) investment and (**b**) comparison of the optimal process of different membrane type combination under the condition of 96% CO_2_ purity and 90% CO_2_ recovery.

Under the condition of the unfixed membrane types Section 3.2 and Section 3.3, the optimization result shows the optimal process is the two-stage CM+HM process with recycle. Then, the processes with the combination of the other three membrane types (CM+CM, HM+HM and HM+CM) are optimized. The optimal process is shown in Figure 13 under the condition of 96% CO_2_ purity and 90% CO_2_ recovery, and the detailed differences of the operating cost and investment cost of these four processes are shown in Figure 14.

As shown in Figure 13a, under the condition of 96% CO_2_ purity and 90% CO_2_ recovery, the optimal CM+HM process has obvious advantages over other processes in capture cost. From Figure 14a, it can be seen that the capture costs of the four processes are mainly affected by the investment and electricity costs. From Figure 14b, it can be seen that compressor and membrane costs account for the major investment costs, and the optimal cost is due to the trade-off between membrane investment cost and power consumption cost. On the one hand, the CM+HM process can save the membrane area when the CO_2_-selective membrane is used in the first-stage membrane (high permeance) and the H_2_-selective membrane is used in the second-stage membrane (high selectivity). On the other hand, the hydrogen concentration in the feed gas accounts for the largest proportion, and when the hydrogen product is obtained from the residue side of the first stage CO_2_-selective membrane, no additional compression process is needed, which can effectively save power consumption.

As shown in Figure 13a,b, compared with CM+HM process, the CM+CM process has higher CO_2_ selectivity and permeability, which leads to lower membrane investment cost, as shown in Figure 14b. Hydrogen is obtained from the residue side of first-stage membrane. However, the CO_2_ product was twice compressed in the inlet and permeate of the second CM, causing an extra 15.7 MW of power and more operating costs in CM+CM. As shown in Figure 13b, the two-stage H_2_-selective membrane process is similar to the two-stage CO_2_-selective membrane process, but the difference is that it takes extra power to compress the H_2_ product to 3000 kPa. At the same time, because the permeate side flow rate in the first-stage membrane is high and the permeance of hydrogen is low in H_2_-selective membrane, the first-stage membrane area reached 111,000 m^2^, so the total membrane area also increased greatly, as shown in Figure 13c.

It is worth noting that, as shown in Figure 13d, in the HM+CM process, although the H_2_/CO_2_ product is also obtained from the residue side, it has the highest capture cost. On the one hand, similar to the two-stage H_2_-selective membrane process, the first-stage membrane of HM+CM process has a higher total membrane area when the H_2_-selective membrane is used, because the permeate flow rate of the first-stage membrane reaches 20,740 kmol/h. On the other hand, because of the high hydrogen concentration in the syngas, the higher flow rate on the permeate side of the first-stage membrane when the first-stage membrane is the H_2_-selective membrane also leads to higher power consumption before entering the second-stage membrane and when the H_2_ product is compressed to 3000 kPa, which leads to the highest capture cost, as shown in Figure 13d.

**Figure 15:**
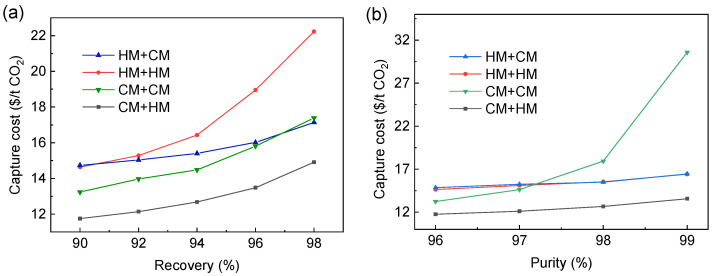
Cost of different membrane combination processes (**a**) vary with CO_2_ recovery when CO_2_ purity is 96%; (**b**) vary with CO_2_ purity when CO_2_ recovery is 90%.

How the CO_2_ capture cost of different membrane combinations varies with CO_2_ purity and recovery is shown in Figure 15. As shown in Figure 15a, when the CO_2_ purity is constant, with the increase of CO_2_ recovery, the cost of single membrane type combination (HM+HM, CM+CM) will increase more obviously; especially when the CO_2_ recovery is high, the use of two-stage H_2_-selective membrane will lead to a large increase in cost. As shown in Figure 15b, when the CO_2_ recovery is constant, with the increase of CO_2_ purity, the cost of the CM+CM process increases remarkably, while the combination of other membrane types is basically unchanged. When the CO_2_ purity is 99%, the cost of the CM+CM process is 30.58 $/t CO_2_. Therefore, it is uneconomical to use CM+CM to obtain high-purity CO_2_ even if the CO_2_-selective membrane has excellent performance.

**Figure 16:**
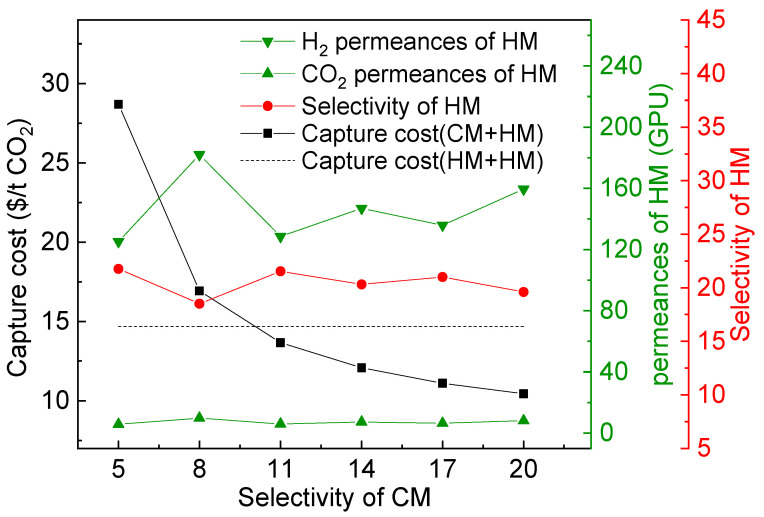
Variation of the cost of the CM+HM process and the best separation performance of HM with the change of the selectivity of CM.

It should be noted that Figure 15 is the result under the upper bound of the CO_2_-selective membrane. If the selectivity of the CO_2_-selective membrane changes, the best combination of membrane types may change, as shown in Figure 16. Under the condition of 96% CO_2_ purity and 90% CO_2_ recovery, when the selectivity of the CO_2_-selective membrane changes, the optimal selectivity of the H_2_-selective membrane basically is maintained at 21. When the selectivity of the CO_2_-selective membrane is lower than 10, the cost of CM+HM process is gradually higher than that of the HM+HM process.

### 3.5. Comparison of Different Situations

From Section 3.2 to Section 3.3 we present four different situations:

**Situation 1**: Fixed pressure based on commercial H_2_- and CO_2_-selective membranes.

**Situation 2**: Pressure optimization based on commercial H_2_- and CO_2_-selective membranes.

**Situation 3**: Pressure optimization based on the commercial CO_2_-selective membrane and H_2_-selective membrane performance changes based on the Robeson upper bound.

**Situation 4**: Optimization of both the H_2_ and CO_2_ membrane performance change based on the Robeson upper bound.

**Figure 17:**
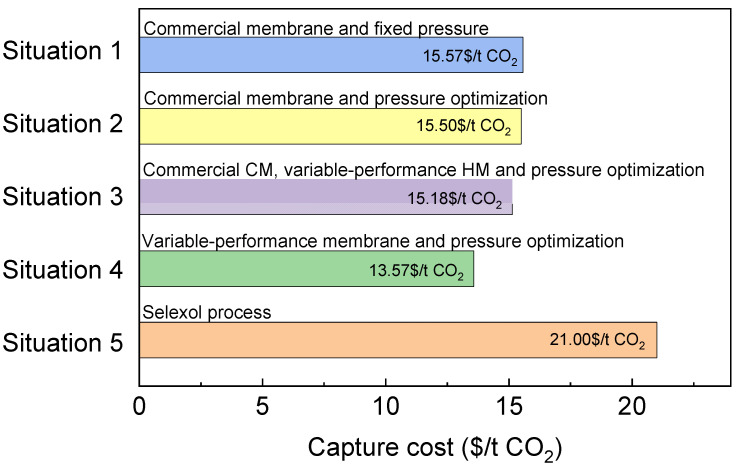
Impact of different optimization on cost in different situations.

To compare with the Selexol technology, the comparison results under different conditions with 99% CO_2_ purity are shown in Figure 17. In addition, the total power consumption and membrane area in each situation are shown in Table 8.

As shown in Figure 17 and Table 8, under the condition of fixed pressure for situation 1, the capture cost is 15.57 $/t CO_2_ by using commercial membrane. In situation 2, by optimizing the operating pressure, the energy consumption is increased by 0.1 MW, but the membrane area is reduced from 77,770 m^2^ to 71,800 m^2^. It effectively saves the investment cost; thus, the capture cost is reduced to 15.50$/t CO_2_. Then, in situation 3, when the performance of the H_2_-selective membrane changes according to Robeson upper bound, although the membrane area is increased, the energy consumption is reduced by 5.6 MW compared with situation 2, and therefore, the cost is reduced to 15.18$/t CO_2_. Finally, in situation 4, when the performance of both the H_2_-selective and CO_2_-selective membranes are optimized according to the Robeson upper bound, both the membrane area and the energy consumption are reduced compared with situation 3, and the corresponding cost is 13.57$/t CO_2_.

In the design of the CO_2_ capture process, the absorption method is usually used as the benchmark for comparison. According to the simulation results of Lee et al. [54] for a 500 MW power plant, the capture cost of the traditional Selexol process for CO_2_ treatment is 21 $/t CO_2_, which is close to the goal of 20 $/t CO_2_ of DOE [55]. It can be seen from Figure 17 that under the condition of keeping the capture rate 90%, even if the CO_2_ purity rises to 99%, the cost is about 13.57$/t CO_2_, which shows that the cost of membrane technology is better than that of absorption technology. Compared with absorption technology, membrane technology also has advantages in investment and installation, so with the improvement of the design method and further research in membrane materials, membrane technology has a very broad development prospect.

## 4. Conclusions

In this paper, the superstructure method is used to design a two-stage membrane process to separate CO_2_ from syngas, so as to capture CO_2_ in pre-combustion. Both H_2_-selective and CO_2_-selective membranes are considered; the optimal process under different product conditions is studied with the CO_2_ capture cost as the objective function.

For the commercial membrane and fixed feed pressure, the CM+HM two-stage process showed the best performance under the condition of 96% purity and 90% recovery, because the compression power consumption would be saved when all products are obtained on the residual side; the result showed that the capture cost is 13.50$/t CO_2_. The optimization results based on the feed pressure of each stage of the membrane show that when the inlet feed pressure of the second stage membrane is further increased, the increase of driving force reduces the total membrane area of the system, and since CO_2_ is obtained from the residual side of the second stage membrane, the power consumption of the system is basically unchanged, and the capture cost can be reduced to 13.41 $/t CO_2_.

When the H_2_-selective membrane performance changes based on the Robeson upper bound, the optimization results show that the capture cost of the optimal process is reduced to 13.22 $/t CO_2_, and the optimal selectivity of H_2_-selective membrane is increased to 20. Under this condition, the power consumption of the system is reduced at the cost of increasing the membrane area, and the capture cost is minimized by balancing the investment and operation cost. The change of the optimal process is studied when the H_2_-selective and CO_2_-selective membranes change based on the Robeson upper bound. The optimization results show that although the CO_2_-selective membrane performance with a selectivity as high as 15.00 and CO_2_ permeance of 2643 GPU is considered, the optimal process does not change with the improvement of CO_2_-selective membrane performance, and the CM+HM two-stage process is still the optimal process. The simultaneous increase of the CO_2_-selective membrane’s selectivity and permeability leads to the decrease of membrane area and power consumption, and the capture cost is further reduced to 11.75 $/t CO_2_.

The above results show that although some product conditions and membrane properties have changed, the optimal combination of membrane types is still CM+HM. The CO_2_ capture cost embodies the trade-off between membrane module cost and power consumption cost (including compressor investment cost and power consumption cost). When the membrane performance changes based on the Robeson upper bound, the capture cost will obviously decrease. Because the CO_2_-selective membrane can improve the permeability and selectivity synchronously, which will greatly reduce the capture cost of the process, it still needs to be used in combination with the H_2_-selective membrane, because obtaining high-pressure products on the residual side will effectively save the power consumption of the system.

Finally, compared with the Selexol method, the results show that the membrane process still shows excellent performance even under the requirement of 99% high CO_2_ purity. This is mainly because the syngas is at a high pressure and the CO_2_ concentration is above 30% in the pre-combustion CO_2_ capture process; the large driving force saves the compression power consumption of the product.

## Figures and Tables

**Table 1:** The range of control variables.

Control Variable		Variable Type	Range
Selection and performance of membrane types	1-stage membrane type selection coefficient x_1_	Binary	{0, 1}
	2-stage membrane type selection coefficient x_2_	Binary	{0, 1}
	1-stage membrane selectivity x_3_	Continuous	[2, 30] for HM [5, 15] for CM [2, 30] for HM [5, 15] for CM
	2-stage membrane selectivity x_4_	Continuous	
	1-stage membrane area x_5_	Continuous	[500, 200,000]
	2-stage membrane area x_6_	Continuous	[500, 200,000]
Process structure	1-stage membrane recycle stream selection coefficient x_7_	Binary	{0, 1}
	1-stage membrane recycle percentage x_8_	Continuous	[0, 100]
	2-stage membrane feed side selection coefficient x_9_	Binary	{0, 1}
	2-stage membrane feed percentage x_10_	Continuous	[0, 100]
	1-stage membrane feed selection coefficient from 2-stage membrane x_11_	Binary	{0, 1}
	1-stage membrane feed percentage from 2-stage membrane x_12_	Continuous	[0, 100]
	2-stage membrane recycle stream selection coefficient x_13_	Binary	{0, 1}
	2-stage membrane recycle percentage x_14_	Continuous	[0, 100]
System parameters	1-stage membrane inlet pressure x_15_	Continuous	[105, 5000]
	1-stage membrane outlet pressure x_16_	Continuous	[20, 105]
	2-stage membrane inlet pressure x_17_	Continuous	[105, 5000]
	2-stage membrane outlet pressure x_18_	Continuous	[20, 105]

**Table 2:** Performance of commercial membrane [4].

Membrane Type	Unit	H_2_ Permeance	CO_2_ Permeance
H_2_-selective membrane	GPU	300	20
CO_2_-selective membrane	GPU	85	1000

**Table 3:** Economic calculation parameters.

Cost Parameter	Unit	Equation	Reference
Membrane module	$	$I_{m}=240\times A_{m}$	[44]
Membrane frame	$	$I_{\mathrm{mf}}=2,380,000\times {\left(A_{m}/2000\right)}^{0.7}$	[45]
Compressor	$	$I_{\mathrm{cp}}=670\times W_{c}$	[37]
Vacuum pump	$	$I_{\mathrm{vp}}=1341\times W_{\mathrm{vp}}$	[37]
Expander	$	$I_{\mathrm{ex}}=500\times W_{e}$	[37]
Heat exchanger	$	$I_{\mathrm{he}}=3,500,000\times L_{\mathrm{he}}/440$	[46]
Annualized capital cost	$	$C_{acc}=\left(I_{cp}+I_{vp}+I_{ex}+I_{he}+I_{mf}\right)\times d+I_{m}\times d_{m}$	[46]
Depreciation factor of other equipment	d	0.064 (25 years)	[46]
Depreciation factor of membrane	d_m_	0.225 (5 years)	[46]
Maintenance cost	$	$C_{mc}=0.036\times \left(I_{cp}+I_{vp}+I_{ex}+I_{he}\right)+0.01\times \left(I_{m}+I_{mf}\right)$	[46]
Electricity cost	$	$C_{\mathrm{ec}}=8000\times 0.05\times \left(W_{c}+W_{\mathrm{vp}}-W_{e}\right)$	[37]
Total cost	$	$C_{total}=C_{acc}+C_{mc}+C_{ec}$	[46]
Cost per ton CO_2_	$/t	$Cost=C_{total}/m_{CO_{2}}$	[46]

**Table 4:** Information on the optimal process of increasing CO_2_ recovery when the H_2_-selective membrane changes based on the Robeson upper bound.

CO_2_ Recovery (%)	90	92	94	96	98
Optimal selectivity for HM	19.85	20.68	20.33	19.27	18.55
Optimal permeance for HM (GPU)	154.9	141.0	146.6	165.8	181.1
1-stage pressure (kPa)	2983	2961	3001	2918	2994
2-stage pressure (kPa)	3908	3919	4025	3964	3796
1-stage area (m^2^)	18,290	20,180	22,200	26,460	31,690
2-stage area (m^2^)	47,850	55,820	56,020	55,220	60,000

**Table 5:** Information of the optimal process of increasing CO_2_ purity when the H_2_-selective membrane changes based on the Robeson upper bound.

CO_2_ Purity (%)	96	97	98	99
Optimal selectivity for HM	19.85	19.84	20.03	19.74
Optimal permeance for HM (GPU)	154.9	155.2	151.7	156.9
1-stage pressure (kPa)	2983	2982	2997	2890
2-stage pressure (kPa)	3908	3975	3948	4050
1-stage area (m^2^)	18,290	18,580	18,830	20,220
2-stage area (m^2^)	47,850	53,500	64,790	76,510

**Table 6:** Information of the optimal process of increasing CO_2_ recovery when the H_2_-selective and CO_2_-selective membranes change based on the Robeson upper bound.

CO_2_ Recovery (%)	90	92	94	96	98
Optimal selectivity for CM	15	15	15	15	15
Optimal permeance for CM (GPU)	2643	2643	2643	2643	2643
Optimal selectivity for HM	20.70	20.68	19.77	19.43	18.35
Optimal permeance for HM (GPU)	140.6	140.9	156.3	162.7	185.5
1-stage pressure (kPa)	2948	2995	2984	2968	2995
2-stage pressure (kPa)	4001	4232	4054	3878	3636
1-stage area (m^2^)	6923	7440	8347	9668	11,810
2-stage area (m^2^)	44,840	44,750	45,810	50,800	54,390
Specific energy(GJ/tCO_2_)	0.6685	0.6940	0.7261	0.7702	0.8543
Specific membrane area (m^2^/(t CO_2_/h))	115.1	113.5	115.3	126.0	135.2
Capture cost ($/t CO_2_)	11.75	12.14	12.68	13.48	14.91

**Table 7:** Information of the optimal process of increasing CO_2_ purity when the H_2_-selective and CO_2_-selective membranes change based on the Robeson upper bound.

CO_2_ Recovery (%)	96	97	98	99
Optimal selectivity for CM	15	15	15	15
Optimal permeance for CM (GPU)	2643	2643	2643	2643
Optimal selectivity for HM	20.70	20.43	19.31	20.18
Optimal permeance for HM (GPU)	140.6	144.9	165.0	149.2
1-stage pressure (kPa)	2948	3000	2936	2938
2-stage pressure (kPa)	4001	4080	4104	4363
1-stage area (m^2^)	6923	6871	7209	7376
2-stage area (m^2^)	44,840	49,210	51,330	67,550
Specific energy(GJ/tCO_2_)	0.6685	0.6872	0.7179	0.7570
Specific membrane area (m^2^/(t CO_2_/h))	115.1	124.7	130.2	166.6
Capture cost ($/t CO_2_)	11.75	12.11	12.66	13.57

**Table 8:** Comparison of different situations under the conditions of 99% purity and 90% recovery.

System Parameter	Situation 1	Situation 2	Situation 3	Situation 4
1-stage membrane permeance	α_CO2/H2_ = 11.76 J_CO2_ = 1000 GPU	α_CO2/HF_ = 11.76 J_CO2_ = 1000 GPU	α_CO2/H2_ = 11.76 J_CO2_ = 1000 GPU	α_CO2/H2_ = 15.00 J_CO2_ = 2643 GPU
2-stage membrane permeance	α_H2/CO2_ = 15 J_H2_ = 300 GPU	α_H2/CO2_ = 15 J_H2_ = 300 GPU	α_H2/CO2_ = 19.74 J_H2_ = 156.9 GPU	α_H2/CO2_ = 20.18 J_H2_ = 149.2 GPU
Number of compressor	3	4	4	4
Number of expander	0	1	1	1
Power consumption of compressor (MW)	109.7	110.0	104.8	94.9
Output power of expander (MW)	0	0.2	0.6	0.3
Net power consumption (MW)	109.7	109.8	104.2	94.6
Membrane area (m^2^)	77,770	71,800	96,730	74,926
Capture cost ($/t CO_2_)	15.57	15.50	15.18	13.57

## Data Availability

The data presented in this study are available on request from the corresponding author.

## Nomenclature

Symbols	
x	optimization variable
*α*	selectivity
*P_i_*	permeability of the fast gas i
*k*	front factor
*n*	slope of the log–log plot of the Robeson upper bound
*L*	flow rate of heat exchange stream
*r*	penalty factor
Subscripts	
*m*	membrane
*mf*	membrane frame
*cp*	compressor
*vp*	vacuum pump
*ex*	expander
*he*	heat exchanger
*acc*	annualized capital cost
*mc*	maintenance cost
*ec*	electricity cost
*spe*	specific
*cap*	capture
Acronyms	
IGCC	integrated gasification combined cycle
CM	CO_2_-selective membrane
HM	H_2_-selective membrane
F	feed stream
P	permeate side stream
R	residue side stream
MINLP	mixed-integer nonlinear programming
*I*	investment
*A*	membrane area
*W*	power consumption
*C*	cost
